# Space Compositional Aspects Regarding the Importance of Trees in the Urban Landscape

**DOI:** 10.3390/plants12132581

**Published:** 2023-07-07

**Authors:** László Zoltán Nádasy, István Valánszki, Máté Sárospataki

**Affiliations:** 1 Department of Landscape Protection and Reclamation, Institute of Landscape Architecture, Urban Planning and Garden Art, Hungarian University of Agriculture and Life Sciences, 1118 Budapest, Hungary; nadasy.laszlo.zoltan@uni-mate.hu (L.Z.N.); valanszki.istvan@uni-mate.hu (I.V.); 2 Department of Garden Art and Landscape Design, Institute of Landscape Architecture, Urban Planning and Garden Art, Hungarian University of Agriculture and Life Sciences, 1118 Budapest, Hungary

**Keywords:** heritage protection, historic gardens, visibility, tree compositions

## Abstract

Individual trees and tree compositions provide a wide range of cultural ecosystem services, including playing a key role in defining urban character. In Hungary, urban landscape protection tools have recently been expanded, bringing the topic into the spotlight. However, the significance of natural elements (and particularly trees) in relation to the urban landscape is still under-researched. In this paper, using a novel methodology, the character-forming significance of trees and tree-compositional elements of historic gardens in Hungary that define the urban character is analysed and evaluated. The urban landscape protection tools that establish the current recognition of green elements within the urban landscape are also analysed. In addition, the spatial situations and characteristics making certain trees in historic gardens defining character elements within Hungarian settlements are studied. Reasons behind the lack of significant tree features in certain historic gardens, as well as the external and internal characteristics of tree elements that determine their visual impact have been categorised. The results reveal that visually important trees, while diverse, show distinct trends in terms of visibility and are subject to constant change. The results imply that a paradigm shift is necessary to maintain, design and regulate green infrastructure in relation to visually important trees.

## 1. Introduction

Ecosystem services provide necessary and beneficial services for human well-being [1,2]. Several types of classification exist, however, with the most common being: provisioning services, supporting services, regulating services and cultural services [1,3]. Cultural ecosystem services (CES) can be interpreted as those non-material benefits obtained from ecosystems through recreation, aesthetic experiences, spiritual enrichment, cognitive development and reflection [1]. They have a significant effect on human well-being and life quality. In addition to other ecosystem services, they are also important in every society, and public awareness of them urgently needs to be raised [4].

Thanks to the many disciplines dealing with CES, their meaning and interpretation differ according to geographical location, as well as socio-cultural and professional background [3,5,6]. Following the same line, several classifications exist, but the most commonly used are the following categories: spiritual and religious, recreation and ecotourism, aesthetic, inspirational, educational, a sense of place, and cultural heritage. In this research, the effect of urban trees, particularly trees located in historic gardens within the urban landscape and character, is part of the aesthetic-perceptual and also cultural heritage categories [1]. The Millennium Ecosystem Assessment also emphasised the lack of recognition of CES in landscape and urban planning as well as in heritage protection.

Certain ecosystem services provided by trees are well-known and have been documented. However, cultural ecosystem services and the role trees play in building local character and the urban landscape in particular, have been a relatively under-researched topic, especially compared with regulating and supporting ecosystem services. To combat the relative lack of knowledge regarding cultural ecosystem services provided by trees in historic settings, this study focuses on their visual importance and value to the urban landscape.

Trees, especially trees in historic settings, can become objects of attachment and pride for locals, as well as being easily identifiable features for visitors. Individual trees can have myths and legends attached to them, and they may be prominent features in people’s memories and mental maps. In many ancient cultures, mature trees were treated as gods. They were personalised, they continue to play a role within legends and may have religious significance. Legendary species were admired and protected. In some cases, trees could obtain attention because they were planted in an important place in history or they have connections with important people [7]. These kinds of trees can be located outside of settlements (such as General Sherman, the largest tree in the world, or the Linden of Zsennye in Hungary), or within them.

This research is focused on trees and compositional elements consisting of trees in Hungarian historic gardens and their urban context, especially their impact on the urban landscape as an ecosystem service. The definition of “*historic garden*” was formulated by the ICOMOS-IFLA International Committee for Historic Gardens in 1982 [8]. The Charter makes clear historic gardens’ significance in terms of heritage and the primary role of vegetation as a “*living monument*” feature in the spatial composition of these gardens. Historic gardens are not only of value in and of themselves but also in terms of the garden architectural elements they contain. When historic gardens are located within an urban environment, their tree species may also be recorded in people’s mental maps as a characteristic element of a settlement or part of a settlement; hence, an individual tree, group of trees or row of trees cannot only be part of a historic garden but also contribute to the character of the whole urban landscape, providing (cultural) ecosystem services. In this paper, the focus of analysis is how trees located in historic parks and gardens impact the local landscape and how they can become central elements of the urban character.

This paper focuses on trees within Hungarian historic parks and gardens and the effect of such trees on the settlement’s landscape. Therefore, selected gardens and parks with notable individual trees that are located within or very close to the urban environment were selected—gardens and parks with no visual connection to settlements (such as the gardens of Rum and Alcsútdoboz) are not included in this current research. The factors enabling and increasing the effect of individual trees on the urban landscape, including location and contrast, are studied, as well as reasons that can cause otherwise notable trees (large solitary trees in a suitable situation) to lose their importance and cultural ecosystem service.

In the last decade, Hungary has introduced new regulatory and planning tools for the protection of the urban character and landscape—the system of Urban Landscape Handbooks (*Településképi Arculati Kézikönyv*) and municipal decrees for the protection of the urban landscape (*településkép védelméről szóló rendelet*) has been in force since 2017. As every municipality had to create one of each document, the framework for the protection of local visual character and heritage can be considered complete. However, the handbooks and decrees effectively created and accepted by local municipalities almost exclusively focus on built heritage and architectural character, and rarely mentions the elements of green infrastructure, trees and other plants, even though the official framework allows their inclusion as protected visual elements [9].

In this research, the effect of trees located in gardens and parks of historical value on the urban environment and townscape is analysed. As the focus is on the public visual perception of these plants and the mental connections, the attachment people feel towards them, only trees that are located in publicly accessible open spaces or are visible from public areas (streets, parks) at eye level were taken into consideration.

There are numerous international examples of manuals, handbooks and other documents created to help protect the local urban landscape, especially in Latin America (Buenos Aires, Colima, Estado de México, Zapotlán el Grande) [10,11,12,13]. However, the topic of trees as valuable and important elements of the urban landscape is barely mentioned in any existing manuals. In cities that have a category for locally important protected or “heritage trees” (Quito, Portland, Cuenca, Budapest), their visibility or impact on the urban landscape is not considered a primary factor in selecting such trees [14,15,16,17]. This means that even though the framework for researching and protecting trees with a profound impact on the urban landscape already exists in several places worldwide, there are no examples of it being used in this way.

Based on the above, the following research questions were formulated:

- What are the properties of trees located within historic gardens as compositional elements that make them attract and hold attention, and thereby make them significant elements of the urban environment?
- How big is the area of impact these trees have in terms of visual importance?
- What is the role of seasonality and how much does it determine trees’ visual significance?
- How can trees, as important elements of the urban landscape, be integrated into the existing legal framework and how can their appearance be protected?

## 2. Materials and Methods

There are around 1500 historic gardens and parks in Hungary, according to certain sources [18]. However, the number of gardens with considerable value is much smaller. Therefore, to select and analyse gardens, a database including a more manageable number of such parks was chosen—specifically the list of gardens from a piece of research conducted previously (Dendrological gardens in 19th-century garden architecture in Hungary) concerning a more limited selection of Hungarian landscape-style gardens [19]. Most still existing Hungarian garden heritage elements were constructed or transformed in the late 18th century and the 19th century in the landscape garden style, in part due to their popularity amongst trend-following owners; however, some were demolished for political ends in the second half of the 20th century, but fortunately, a large number of such gardens survived from this period. This research focuses on parks situated in or directly adjacent to the urban fabric, as trees located within these areas can be reasonably expected to have an effect on the local urban landscape.

**Figure 1:**
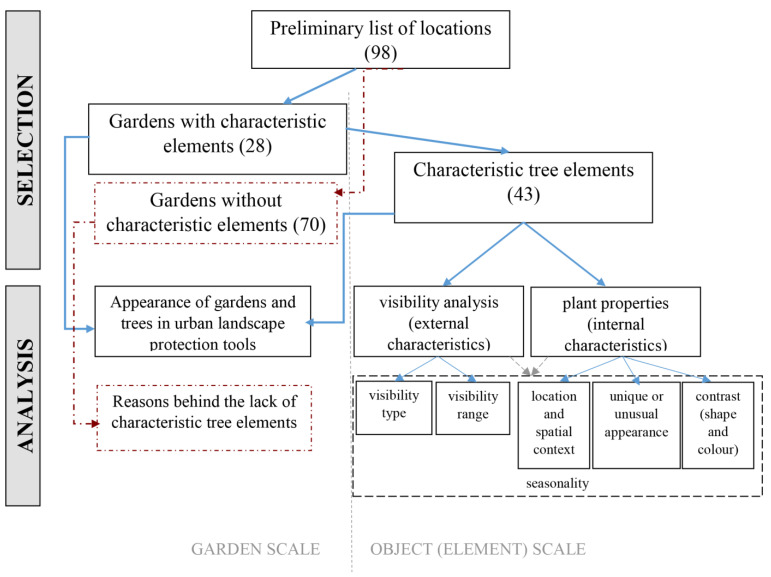
Flowchart of research methods. Blue arrows represent research regarding gardens with characteristic tree elements. Red dotted arrows represent research regarding gardens without such elements. Grey dotted arrows represent research on seasonality.

As established in the Introduction, trees as urban landscape elements are rarely included in existing urban landscape protection tools (handbooks and regulations). Similarly, the impact of trees on the character of the local settlement is a relatively new direction in research. While the perception of trees and attachment towards them have been studied by an increasing number of researchers [20,21,22], these typically ignore their urban context. Recent studies [23,24] have focused on individual trees in residential and heavily urbanised environments. The novel research methodology (Figure 1) presented here uses the results of these studies, especially the factors identified as major influences in the visual importance of individual trees (location and spatial context, unique or unusual appearance, contrast) in the analysis.

In this paper, the impact tree elements located in gardens of historic value have on the urban landscape is studied. Therefore, parks and gardens located outside of the urban fabric or only loosely attached to their settlements have been excluded from the analysis. Examples of such gardens include Rum (Bezerédj-Széchenyi chateau garden) and Alcsútdoboz (the park of the summer residence of Palatine Joseph and Archduke Joseph) and several other locations [25,26,27]. Historic gardens with a strong connection to the urban fabric, however, not only have a positive effect on urban ecology, urban climate and the livability of the surrounding settlement but also act as an important element of the local urban character (cultural ecosystem service), even becoming important local landmarks (e.g., Schönbrunn (Austria); Derby (UK); Versailles (France); Fertőd (Hungary)) [28,29,30]. Based on the list of gardens of the source material mentioned previously [19] and adjusting it to present a more representative look into existing Hungarian historic gardens, a total of 98 locations were included on the preliminary list. 

The primary criterion was that compositional tree elements situated in parks must be clearly visible and perceptible from public spaces outside the garden itself, in order for them to be considered elements of the urban landscape. Those characteristic compositional elements in historic parks that can only be seen by walking within the park itself can also contribute to the local character and mental image. However, in this research compositional elements with a visual impact restricted to the park itself were not taken into consideration.

**Figure 2:**
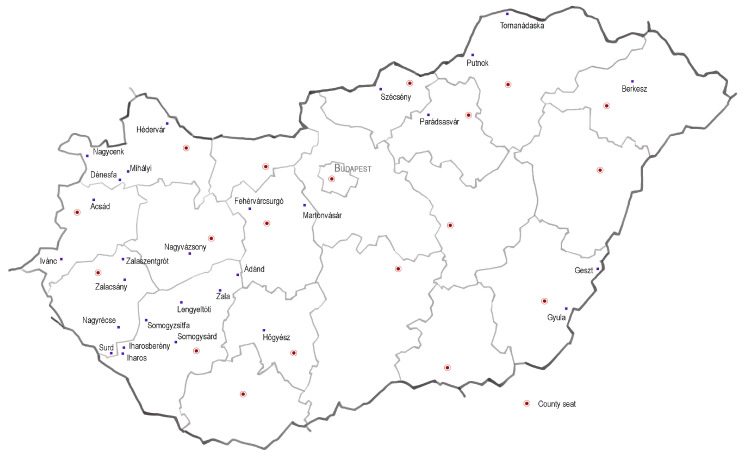
Map of Hungary with selected 28 gardens; map edited by the authors.

The research method (Figure 1) consists of two main parts: selection and analysis. In the selection phase, using the preliminary list of gardens with historic value, a decision was made on whether they include characteristic tree elements (solitary trees, homogenous tree groups or rows of trees) with a significant impact on the settlement’s urban landscape (See Figure 1: Gardens with characteristic elements (28)). All individual tree elements were also listed separately, as a single garden can include multiple characteristic elements. For the selection phase, pictures from field surveys of the past 16 years (since 2007) were used, as well as Google Street View [31]. Ultimately, the final selection reflects the perception and visibility of the study period. The 28 selected sites represent the distribution of historic parks in Hungary well, as there are significantly fewer historic gardens in the Central-Eastern region of the country (Figure 2), for historical reasons. The gardens were created between the 1790s and the 1910s, and the majority of them were constructed in the 19th century.

**Figure 3:**
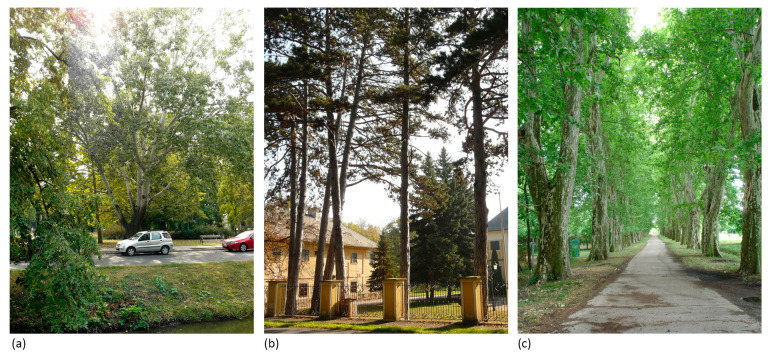
Analysed compositional element types: (**a**) solitary tree—Gyula (2012), (**b**) tree group—Nagyvázsony (2017), (**c**) row of trees—Acsád (2007). Source: M.S.

Within each historic garden, characteristic living compositional elements consisting of trees (solitary tree, tree group, rows of trees) that visually stand out within their environments, and therefore draw attention to themselves with their characteristic appearance, were identified and analysed. Tree elements can be divided into three categories (Figure 3) based on their composition: (1) solitary appearance (standalone individual), which may not only mean a tree located in the centre of a large “empty” (paved or lawn) area but also being a standalone, unique individual amongst a mass of trees of a different species; (2) tree group (of the same species or cultivar), with a visually well-defined, compact form; (3) homogenous rows of trees (of the same species or cultivar).

In the analysis phase, on the garden scale, the inclusion of gardens or their individual tree elements in current municipal urban landscape protection tools as protected or valuable urban elements was studied. In the case of gardens excluded from the selection (those without any tree elements with significant impact), the reasons behind their lack of such elements were studied. In particular, the existence of large, potentially impactful tree elements was evaluated, and in gardens including such trees, the factors behind their lack of visual importance were studied.

On the object scale, the characteristics of selected tree elements (solitary trees, tree groups and rows of trees) that might contribute to their importance were surveyed—their visibility and visual context (external, situational characteristics) and their individual properties (internal characteristics) were analysed separately. In the visibility analysis, elements were categorised based on visibility type (answering the question “How does the tree element appear within the urban landscape?”) and visibility range (answering the question “From what distance is the tree element visible and impactful?”).

**Figure 4:**
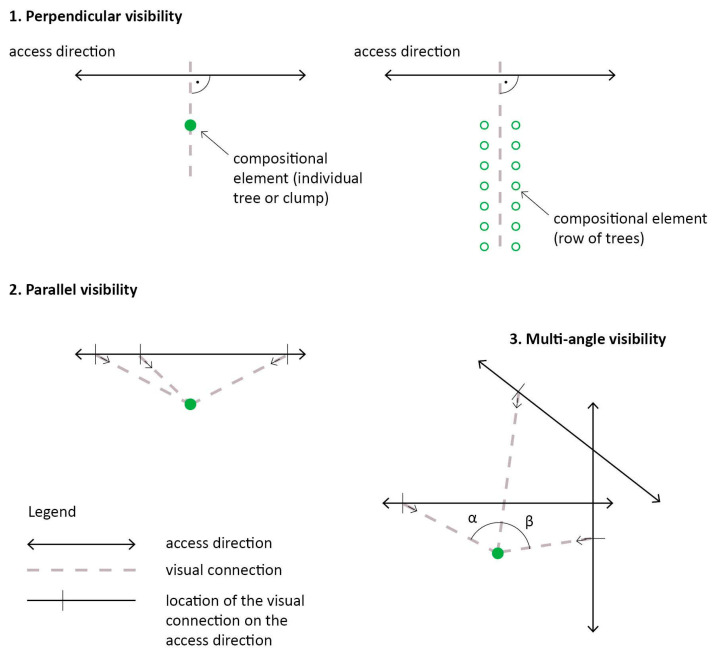
Schematic figures of visibility types (perpendicular, parallel, multi-angle). Source: authors.

Based on field research, the following methodology was developed and applied to describe and categorise the external characteristics. The following three different *visibility types* (Figure 4) can be described:

1. Perpendicular visibility: the character element (tree, tree group or row of trees) is visible at a right angle from the viewer’s path. This type of visibility usually occurs when the element is located close to streets or typical routes of viewers, only becoming visible from a short distance, at a narrow-angle.
2. Parallel visibility: this also occurs when the viewer is on a path close to the character element, but due to the visibility angle being wider, the object is visible from a much greater distance.
3. Multi-angle visibility: the character element is visible and visually impactful from several different viewpoints and directions: each visibility axis is at an angle to all other visibility angles from public spaces, squares, and streets.

In addition to the type of visibility for tree elements, the *distance* within which each of the analysed objects have a significant influence on the urban landscape was also studied. Based on this, visibility range categories of each element within (or even beyond) its urban context were created. Three categories were identified:

A. The characteristic tree element is only visible from a short distance (<50 m)
B. Characteristic elements with a visibility range of an entire neighbourhood or settlement part (>100 m or at least 50 m in several different directions)
C. Elements with visual connections (being perceptible and identifiable) from almost the entire settlement or even beyond the urban borders.

In the case of individual properties (internal characteristics), three plant properties identified by Nádasy (2022) as particularly important contributors to trees’ importance in the urban landscape were analysed: prominent location and unique spatial context (trees located on corners, intersections, in the centre of open spaces, elevated or otherwise focal locations, or those that are much taller than surrounding elements); unique or unusual appearance (compared to the “typical”, single-trunked, symmetrical tree shape) and contrast with nearby elements (including colour and shape contrast, both in terms of foliage and habit). Seasonality (leaf coloration, deciduousness, seasonal features such as flowers or fruits) was also taken into account as a factor in all object-scale analyses.

## 3. Results

### 3.1. Results of the Selection Phase

**Figure 5:**
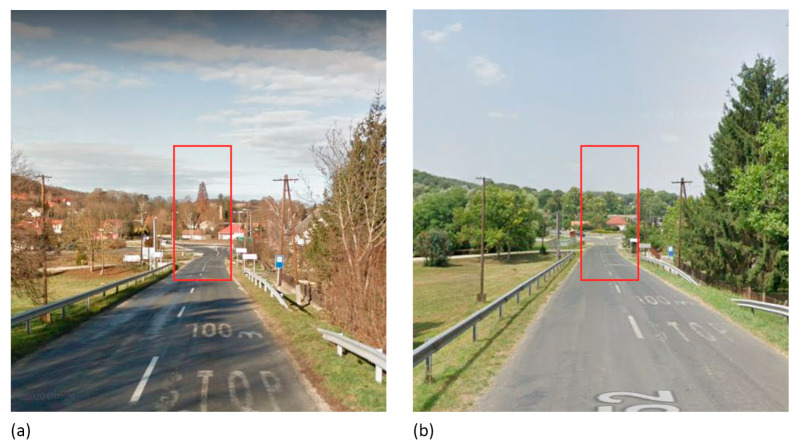
Characteristic compositional elements that once existed in Zalacsány. Source: Google Street View [32]: (**a**) 2011, (**b**) 2021; pictures edited by the authors.

The preliminary list of gardens, containing 98 historic gardens, was narrowed down to a list of 28 parks (Table A1) with at least one characteristic compositional element (individual tree, tree group or row of trees). Ten locations include more than one such element, with the total number of characteristic compositional tree features being 43. As an exception, the ancient *Sequiadendron giganteum* in Zalacsány has been included on the list, despite its death in the drought year of 2012, as it was an exceptionally important feature of the settlement’s image and a major landmark (Figure 5), even though it is not physically present anymore. In the selection phase, any gardens located separately from the settlement that are not included in the urban fabric itself (e.g., the well-known Alcsútdoboz arboretum) were removed, as these gardens do not have enough urban context to be included in the detailed analysis.

### 3.2. Results of the Analysis Phase

The analysis phase involved two different scales, the garden and object scale, as mentioned in the Section 2. Results will therefore be presented following the same scale-based categorisation.

#### 3.2.1. Results of the Garden Scale Analysis

The reasons why gardens included in the preliminary list do not have characteristic tree elements with a marked impact on the urban landscape were analysed. The reason for excluding gardens from the second phase that are directly connected with the neighbouring settlement was that there is no characteristic, unique tree elements that have a significant impact on the urban landscape. Several factors were identified that may result in a lack of characteristic tree elements in historic parks.

**Figure 6:**
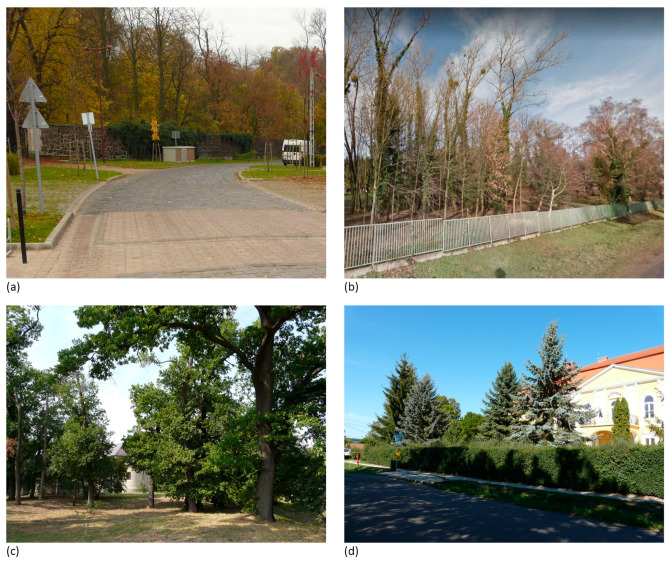
Homogenous or mass-like tree cover without conspicuous elements: (**a**) Vácrátót—2013; (**b**) Vép—2012; (**c**) Doboz—2012; (**d**) Názsa—2012. Source: **a**, **c**, **d**: M.S., **b**: Google Street View [32].

Several gardens currently do not include trees that could potentially be important landmarks. This is mostly due to the homogenous tree cover or a large, uniform mass of trees that does not allow any individual trees or compositional elements to stand out and become notable, unique visual features. Featureless plant mass falls into the same category. Examples include Vép, Doboz, Nádasdladány, Názsa, and Bicske (Figure 6).

In several gardens, existing and potentially visually impactful compositional elements can be identified, but they are not visible from public spaces or their visibility is so reduced that they cannot be considered an important element of the urban landscape. These elements can be hidden from view in several different ways, by plants, built elements or topography.

**Figure 7:**
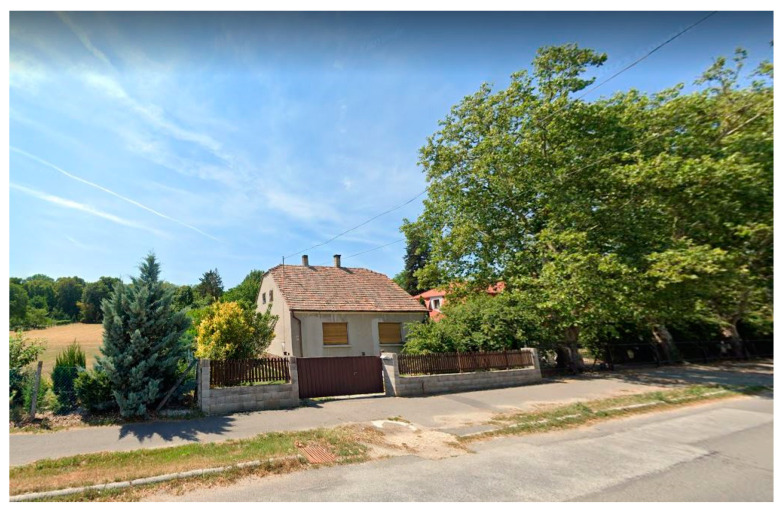
Buildings and green elements hiding the garden of Szeleste from view (2022). Source: Google Street View [32].

**Figure 8:**
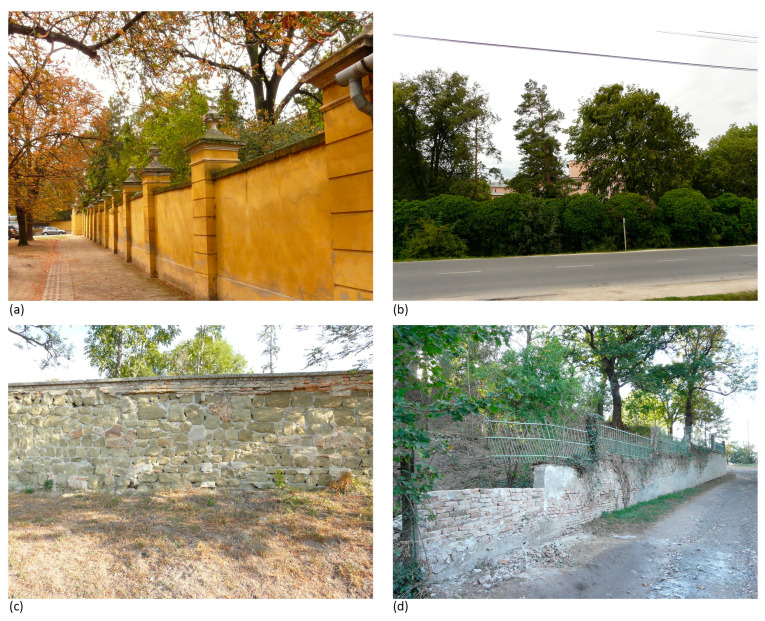
Built elements restricting the visibility of parks in Ikervár (**a**)—2010, Kalocsa (**b**)—2012 and Acsa (**c**,**d**)—2012. Source: M.S.

- Topography can hide even the largest trees if they are located behind a hill or mound, or they stand significantly below street level or on top of a steep incline, outside the comfortable view range of the average onlooker. The garden of Szeleste is a good example of tree elements being hidden on different sides of the park by topography, buildings and homogenous green walls (rows of street trees) (Figure 7).
- Living elements can also block characteristic trees from view. Hedges, shrubs and trees (either deliberately planted or spontaneously grown from seed) located around the borders of the property can make anything, including the most conspicuous tree elements, invisible from the outside. An example of this is the historic garden of Körmend, where spontaneously grown vegetation hides most of the notable trees from most public viewpoints.
- Built elements are the most common features restricting the visibility of trees inside historic gardens. Buildings surrounding the garden (e.g., the former archducal park of Sárvár) and tall solid fences (e.g., the park of Batthyány Mansion in Ikervár; the garden of the former Vigyázó Mansion in Vácrátót; the Archbishop’s garden of Kalocsa) can both block elements from view (Figure 8).

Several of the identified factors may also be present at the same time. A good example of this is the garden of the Prónay Mansion in Acsa, where the park’s existing and visually characteristic tree elements are invisible from public areas, due to the combined hiding effect of a tall solid fence, a mass of vegetation around the garden, buildings within the park and the trees being located in an unfavourable location in terms of topography.

The urban landscape protection tools were also analysed in all 28 settlements where gardens with characteristic tree elements with a marked impact on the urban landscape are located (Table 1). Out of the 28 urban landscape handbooks, only 11 (39%) mentioned the importance of the historic garden or its trees as significant elements in the urban landscape or as valuable visual heritage. Good examples include the handbooks of Berkesz and Dénesfa, where both the garden and its trees are emphasised as important, valuable and in need of urban landscape protection, and Tornanádaska, where the giant *Sequoiadendron giganteum* itself is mentioned for its visual appeal.

Seventeen (61%) of the handbooks do not mention the analysed parks at all (Putnok, Lengyeltóti etc.), often only analysing the chateau or castle buildings, but not their surroundings. Others only include it in the enumeration of public green spaces (Gyula, Martonvásár, Mihályi) or as a nature protection area (Hőgyész, Ivánc, Szécsény), but not as valuable elements of the urban landscape. This is especially noteworthy as the majority of settlements included in the analysis are small towns or villages, with relatively few landmarks.

#### 3.2.2. Results of the Object Scale Analysis

##### External Characteristics

**Figure 9:**
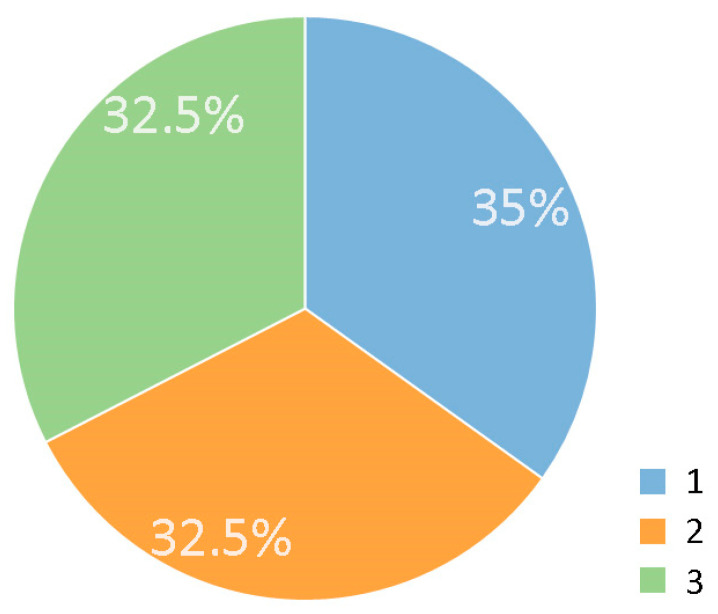
Proportion of visibility types (1, 2, 3). Key: 1. Perpendicular visibility; 2. Parallel visibility; 3. Multi-angle visibility. Source: authors.

The results of the object-scale analysis show that the three different visibility types are similarly common among the 43 analysed elements. Perpendicular visibility can be observed in 15 cases (35%), while 14 elements (32.5%) were visible in a parallel way. Fifteen tree elements (32.5%) are also characterised by multi-angle visibility. The proportional occurrence of all three types demonstrates that all of them can be considered widespread and typical (Figure 9).

**Figure 10:**
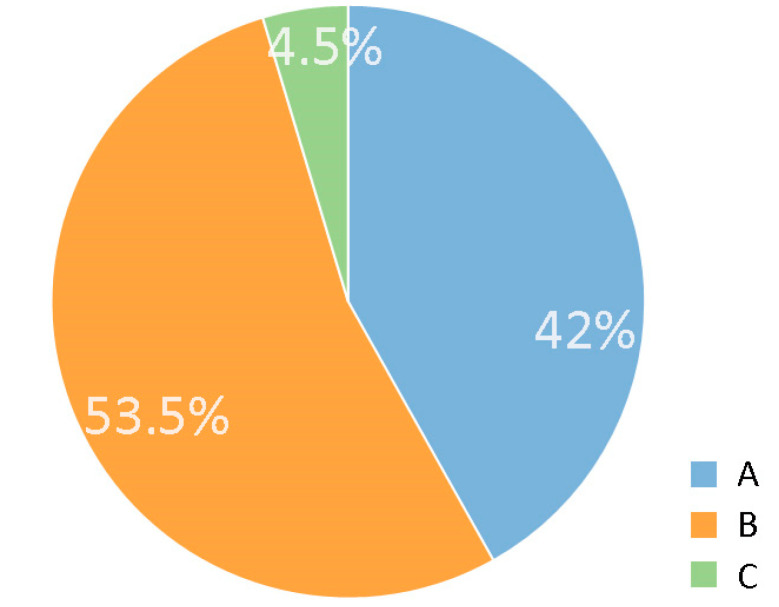
Proportion of the different visibility range types (A, B, C). Key: (A) short distance visibility of characteristic tree elements (<50 m); (B) character element that can be perceived from several directions in the settlement (>100 m or at least 50 m in several different directions); (C) a character element that can be perceived from almost the entire settlement or even beyond the urban borders. Source: authors.

In terms of visibility range, there are much larger differences than in the case of visibility types (Figure 10). 18 studied objects (42%) are only visible from a short distance (category A), 23 (53.5%) can be viewed from different parts of the settlement (category B), and only two (4.5%) are visually impactful from beyond their close urban environment (category C).

Seven different combinations of visibility type and visibility range categories have been recorded (Table 2). The visibility range category C does not have any combinations with visibility types 1 and 2, as these mean there are more localised visual connections, while category C implies a much wider area of visual impact. The most common combination—32.5%, 14 occurrences—was between the perpendicular (narrow) visibility type and small visibility range (1/A). This means that the majority of analysed gardens—and settlements—do not typically have large-scale visual connections, and visibility is often blocked. One of the second most common combinations (2/B), with 25.6% of all cases (11 examples), is also logical from a visual design standpoint: a linear visibility axis can often extend over 100 m. The combination 3/B, which also occurs in 25.6% of examples (11 occurrences) included in the analysis, is similar to 2/B, but with multiple angles of visibility. 

The combination 1/B was identified in a single case (Szécsény), where a solitary *Sophora japonica* ‘Pendula’ is visible from a much larger distance than 50 m, even though the angle of visibility is quite narrow. Three examples (7%) have been found for the combination 2/A (parallel visibility type with a mid-sized range): Iharosberény (*Ginkgo biloba*), Fehérvárcsurgó (*Aesculus hippocastanum*) and Somogysárd (*Sequoiadendron giganteum*). In each of these gardens, the characteristic trees are visible from a path running nearby them, but due to an object (vegetation or buildings) blocking them from view, they only reveal themselves when the visitor is already closer than 50 m.

**Figure 11:**
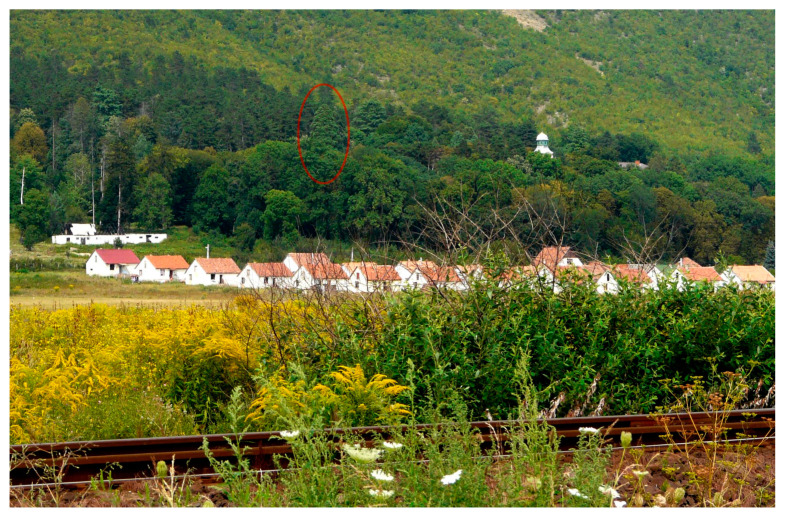
Visual connection beyond the settlement borders. Characteristic giant sequoia in the park of Hadick mansion in Tornanádaska—2012. Source: M.S.

Characteristic elements visible from a wide angle or multiple directions (category 3) include a single example of *Platanus* trees located in the front garden of the Hédervár Castle that are visually dominant in their surroundings, but due to the dense vegetation bordering the garden, they only appear when visitors are already in front of the castle (3/A). This effect is unchanging all year, even in the winter, when there are no leaves. Category 3 also includes tree elements that are visible from an exceptionally large range. The giant sequoia (*Sequoiadendron giganteum*) in Tornanádaska is easily visible from outside the village, making it an important local landmark (Figure 11). Another *Sequoiadendron* specimen in Zalacsány was visible from over 600 m before 2012 (Figure 5). In both examples of the 3/C combination, advantageous topography helped to create a wide visibility range for trees.

##### Internal Characteristics

After categorising the visibility of compositional elements, an analysis was made regarding which inherent properties of trees and tree compositions are the most important factors behind their impact on the urban landscape, as previously explained in the Section 2.

**Figure 12:**
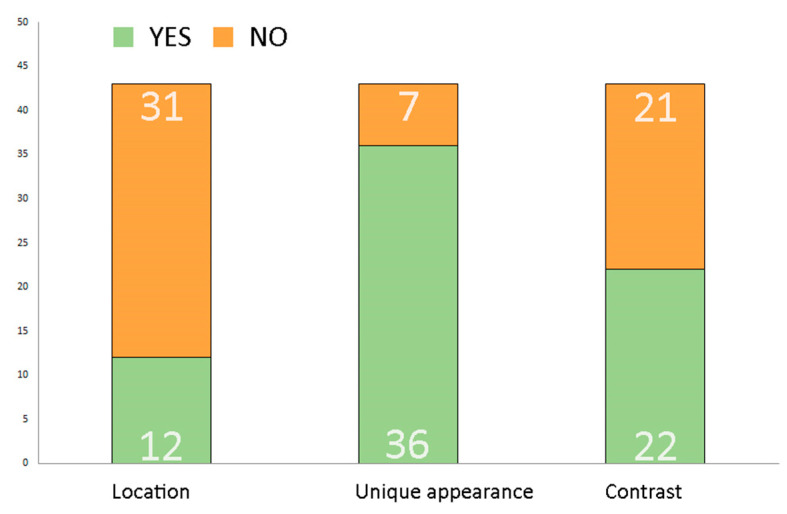
The presence or absence of internal characteristics (location, unique appearance, contrast) of the elements. Source: authors.

According to the analysis results, unique or unusual appearance is the most common factor, with 36 (83%) of studied elements having significantly different shapes or habits from “typical” trees. Contrast with neighbouring living elements, in colour or shape, or even both in some cases, can be observed in 22 (51%) of trees and tree compositions. Trees standing in a prominent, special location (e.g., on corners or in the centre of large open spaces) were the least common, with only 12 (28%) such elements occurring in the analysis (Figure 12).

**Figure 13:**
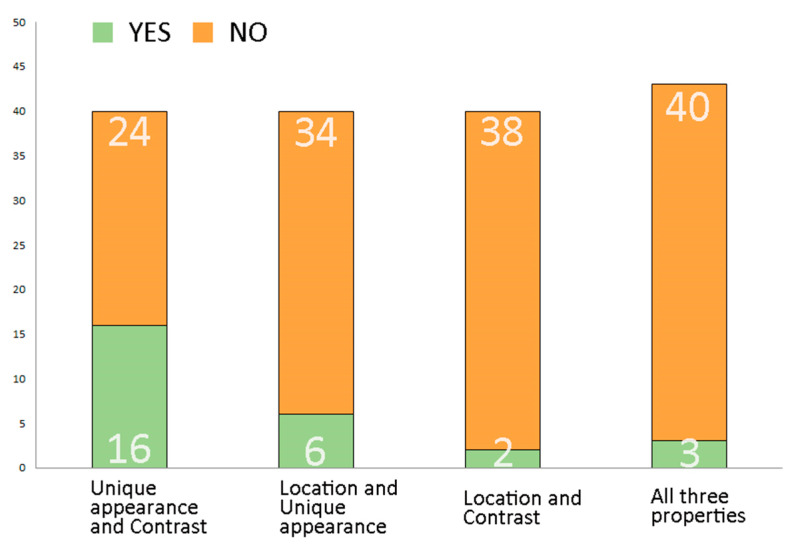
The number of elements with more than one internal characteristic. Source: authors.

The occurrence of different combinations also led to interesting results. The most common combination was between unique appearance and contrast, in 19 cases. Nine tree elements with a unique or unusual appearance and a prominent, special location were observed, while five trees standing in a special location and with a significant contrast to their surroundings were identified (Figure 13).

In three cases, all three analysed factors were present: the poplar (*Populus alba*) of Gyula that stands in a prominent open area next to a road provides visual interest with its multi-stemmed habit, the light-coloured trunks and silver-backed leaves providing contrast with surrounding plants. The same garden includes a group of sycamores (*Platanus* × *hybrida*) that also show all three analysed properties. The third such example is the sycamore (*Platanus* × *hybrida*) in Ádánd, which has a tilted trunk (unique shape) bending over a road (prominent location), with differently coloured trees in its vicinity (contrast). 

In a separate analysis, the way seasonality affects the prominence of trees in the urban landscape was studied. Several trees were especially noticeable during certain seasons: evergreen plants stand out from a mass of deciduous trees during the winter, while they may not even be visible in summer. The trunks, bark colouration, branch structure and unique habits of certain trees (*Platanus* species and *Aesculus hippocastanum* in particular) are also more prominent in leafless periods than during the warmer months. Taxa with spectacular leaf colouration in the autumn (*Ginkgo biloba*, *Larix decidua*) or in spring and early summer (*Fagus sylvatica* ‘Atropunicea’ and other purple-leaved cultivars) are also highly seasonal in appearance, and even though their location, size or habit can also help them become local landmarks, seasonal colouration certainly strengthens the effect.

## 4. Discussion and Conclusions

The results described above have several practical implications for several fields of study, including landscape architecture, green surface maintenance, tree assessment and the study of ecosystem services. Results show that the contribution to the urban landscape is a major component of the cultural ecosystem services of trees, which as a factor is rarely included in currently-used tree evaluation methods [33,34]. Results concerning tree visibility types can be particularly helpful in furthering research on the perception-based value of trees [20,21] and visibility-based research on urban areas [35]. The impact of trees and tree compositions on the urban landscape and therefore the perception and attachment of people to historic gardens within the urban fabric can be a meaningful new approach to research into cultural ecosystem services [3,32,36,37].

When also taking the contribution of trees and tree compositions to the urban landscape into account as a cultural ecosystem service, priorities for maintenance and the design of green areas and vegetation may be somewhat different. In historic gardens and green areas with existing elements that have a major impact on the surrounding urban landscape, retaining—and if possible, improving—visibility must be the most important aspect of maintenance. In other cases, where potentially impactful trees and tree elements are currently hidden from view, the removal of existing features (living or built) that block their visibility from public areas may be an overall positive decision, especially if these features do not have any inherent historic or cultural value. Research results suggest that even the removal of surrounding, less characteristic or valuable trees might be necessary to improve the visibility and visual impact of tree elements. Similarly, the design and execution of green infrastructure development must take into consideration existing trees and tree compositions of value to the urban landscape. Newly-planted street trees, hedges or shrubs may block the visibility of these valuable elements, overall reducing the cultural ecosystem services of their surroundings. This is especially true of tall and dense shrubs and evergreens in particular, as these create an unchanging spatial wall, completely severing visual connections. 

The unique appearance of certain individual trees can be attributed to properties that are generally seen as undesirable in tree assessment and maintenance. Asymmetrical growth, twisted branches and visible scars can all contribute to the uniqueness—and therefore the visual importance—of these plants. While most existing tree assessment methods consider these properties negative and the goal of traditional maintenance is usually to change or “rectify” them, the results of this research imply that there is an inherent value in the unusual and bizarre appearance of certain trees. However, this does not mean that these characteristics must be maintained at all costs: any factors endangering the survival or overall attractiveness of trees, and especially visitor safety, can and should be prioritised over visual interest. 

**Figure 14:**
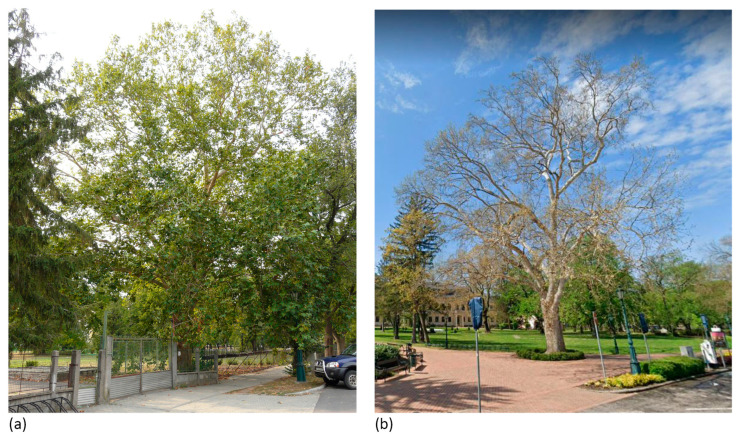
A sycamore tree in changing environment (Gyula). Source: (**a**) M.S.–2012, (**b**) Google Street View [32]—2021.

The results suggest that time is a significant factor in the visual importance of trees. The analysed elements are dynamic, living components of the urban landscape, and their characteristics can change drastically on several different time scales, which can be due to changes in the seasons or even death because of extreme weather conditions, diseases or senescence (Figure 5 and Figure 14). However, due to time restraints, this research could not analyse this complex topic in detail. Still, the results show that the role of trees in the urban landscape may decline or completely cease to be due to many factors, and trees can also become important features over time. Further studies are necessary to fully understand the impact of seasonal changes and long-term processes on the system of visually important tree elements.

Hungary provides a good case study for this research because there is a large number of towns where historic parks account for a significant proportion of their greenery. The European network of connections between members of ruling houses, nobility and the clergy, as well as in scientific and artistic circles, is well known. Through marriage, travel, due to the role of the Church as an intermediary, as well as the invitation and employment of various professionals and craftsmen (stonemasons, botanists, and garden artists), a similar cultural foundation formed throughout the continent, which also allowed the evolution of local, vernacular specialities in garden history, unique to the local landscape [38,39,40,41]. Because of this, the results can potentially be applied to various other European countries. Furthermore, the existence of a fully implemented urban landscape protection system in Hungary provides the necessary framework to successfully study the topic in all its complex contexts. While the methods and results are not specific to the country and can be considered to be widely applicable and globally relevant, further research in different geographical locations and different types of green areas can establish a wider recognition of the importance of trees and tree compositions in the urban landscape.

Research on the inclusion of trees associated with historic gardens and parks in urban landscape protection tools in Hungary suggests that even with an existing framework, the recognition of trees as visually significant elements are lacking. While regulation on the protection of the urban landscape is implemented to a variable degree around the world, professional recognition of features as valuable is key to ensuring their survival and maintenance. Furthermore, results suggest that the visibility and visual connections of trees can also be extremely important. This implies that in addition to ensuring the protection of trees and tree compositions themselves, integrating them into the wider urban and green infrastructure planning system is vital to maintaining the impact of these valuable living elements in their wider context.

In conclusion, the results show that trees associated with historic gardens have a diverse and dynamically changing impact on the urban landscape, which has recently come to play an increasingly important role in protecting local cultural heritage. Factors behind the visual impact on tree elements were identified, as well as different visibility types, which can help categorise and preserve the cultural services provided by these living pieces of cultural heritage.

## Figures and Tables

**Table 1:** Emphasis on gardens and trees in urban landscape handbooks.

Name of Settlement	Tree Elements or Gardens Emphasised in Handbook as Valuable
Acsád	No
Ádánd	No
Berkesz	Yes
Dénesfa	Yes
Fehérvárcsurgó	No
Geszt	No
Gyula	No
Hédervár	Yes
Hőgyész	No
Iharos	Yes
Iharosberény	Yes
Ivánc	No
Lengyeltóti	No
Martonvásár	No
Mihályi	No
Nagycenk	Yes
Nagyrécse	Yes
Nagyvázsony	Yes
Parádsasvár	No
Putnok	No
Somogysárd	No
Somogyzsitfa	Yes
Surd	Yes
Szécsény	No
Tornanádaska	Yes
Zala	No
Zalacsány	No
Zalaszentgrót	No

**Table 2:** Combinations of visibility types and visibility ranges. Key: 1. Perpendicular visibility; 2. Parallel visibility; 3. Multi-angle visibility; (A) short distance visibility of characteristic tree elements (<50 m); (B) character element that can be perceived from several directions in the settlement (>100 m or at least 50 m in several different directions); (C) a character element that can be perceived from almost the entire settlement or even beyond the urban borders.

Visibility Type	Visibility Range	Combination	Number of Examples	% of Examples
1	A	1/A	14	32.5%
1	B	1/B	1	2.3%
1	C	1/C	-	0%
2	A	2/A	3	7.0%
2	B	2/B	11	25.6%
2	C	2/C	-	0%
3	A	3/A	1	2.3%
3	B	3/B	11	25.6%
3	C	3/C	2	4.7%

## Data Availability

Data is contained within the article. The data presented in this study are available in Appendix A.

## Appendix A

**Table A1:** Data table of analysed tree elements. Legend: 1. Perpendicular visibility; 2. Parallel visibility; 3. Multi-angle visibility; (A) short distance visibility of characteristic tree elements (<50 m); (B) character element that can be perceived from several directions in the settlement (>100 m or at least 50 m in several different directions); (C) a character element that can be perceived from almost the entire settlement or even beyond the urban borders.

Nr. of Location	Nr. of Element	Settlement	Name of the Mansion and the Park	Scientific Name of the Trees	Garden Architecture Element	Visibility Type	Visibility Range	Location	Unique Appearance	Contrast
1	1	Ivánc	Park of Sigray mansion	*Sequoiadendron giganteum*	Individual (element)	2	B		x	
2	2	Zalacsány	Park of Malatinszky- Batthyány country house	*Sequoiadendron giganteum* *	Individual	3	C		x	x
3	3	Nagycenk	Park of Széchenyi mansion	*Tilia cordata*	Linear element (row of trees)	1	A		x	
	4	Nagycenk	Park of Széchenyi mansion	*Platanus* × *hybrida*	Linear element (row of trees)	2	B		x	
4	5	Nagyvázsony	Park of Zichy mansion	*Pinus nigra*	Clump (group of trees)	1	A			x
5	6	Tornanádaska	Park of Hadick mansion	*Sequoiadendron giganteum*	Individual	3	C		x	x
6	7	Surd	Zichy Park	*Platanus* × *hybrida*	Linear element (row of trees)	3	B		x	
	8	Surd	Zichy Park	*Quercus robur f. fastigiata*	Individual	3	B		x	x
7	9	Acsád	Park Szegedy mansion	*Platanus* × *hybrida*	Linear element (row of trees)	3	B		x	
	10	Acsád	Park Szegedy mansion	*Sequoiadendron giganteum*	Individual	3	B		x	
8	11	Martonvásár	Park of the Brunszvik mansion	*Sophora japonica ‘Pendula’*	Individual	1	A		x	x
9	12	Iharos	Park of Inkey mansion	*Platanus* × *hybrida*	Individual	2	B		x	
	13	Iharos	Park of Inkey mansion	*Ginkgo biloba*	Individual	1	A		x	x
10	14	Iharosberény	Park of Inkey mansion	*Platanus* × *hybrida*	Mass-like appearance	3	B	x	x	
	15	Iharosberény	Park of Inkey mansion	*Ginkgo biloba*	Individual	2	A			x
	16	Iharosberény	Park of Inkey mansion	*Cedrus* *deodara*	Individual	2	B		x	x
	17	Iharosberény	Park of Inkey mansion	*Fagus sylvatica ‘Atropunicea’* (group of 3)	Clump	2	B		x	x
11	18	Nagyrécse	Park of Inkey mansion	*Sequoiadendron giganteum*	Individual	2	B		x	x
12	19	Somogyzsitfa	Park of Véssey-Somssich mansion	*Quercus robur f. fastigiata*	Individual	1	A	x		x
13	20	Fehérvárcsurgó	Park of Károlyi mansion	*Aesculus* *hippocastanum*	Individual	2	A			x
14	21	Zalaszentgrót	Park of Batthyány mansion	*Platanus* × *hybrida*	Individual	1	A		x	
	22	Zalaszentgrót	Park of Batthyány mansion	*Platanus* × *hybrida*	Individual	2	B		x	
	23	Zalaszentgrót	Park of Batthyány mansion	*Sophora japonica* ‘Pendula’	Individual	3	B		x	x
15	24	Lengyeltóti	Park of Inkey-Zichy mansion	*Platanus* × *hybrida*	Individual	3	B		x	x
16	25	Somogysárd	Park of Somssich mansion	*Seauoiadendron giganteum* (2)	Clump	2	A		x	
17	26	Szécsény	Park of Forgách mansion	*Sophora japonica* ‘Pendula’ *(2)*	Individual elements	1	B	x	x	
18	27	Parádsasvár	Park of Károlyi mansion	*Pinus nigra (2)*	Individual	2	B		x	x
19	28	Geszt	Park of Tisza mansion	*Platanus* × *hybrida (2)*	Individual	1	A	x	x	
20	29	Gyula	Park of Harruckern-Almássy- Wenckheim mansion	*Platanus* × *hybrida*	Individual	3	B	x	x	x
	30	Gyula	Csigakert public park	*Populus alba*	Individual	3	B	x	x	x
21	31	Ádánd	Park of Csapody mansion	*Platanus* × *hybrida*	Individual	2	B	x	x	x
22	32	Hőgyész	Park of Apponyi mansion	*Platanus* × *hybrida*	Individual	2	B	x	x	
23	33	Zala	Park of Zichy mansion	*Aesculus hippocastanum*	Linear element (row of trees)	1	A		x	
24	34	Hédervár	Park of Khuen-Hédervár mansion	*Platanus* × *hybrida* (inside the garden)	Individual (in winter), mass-like appearance (summer)	3	A		x	
	35	Hédervár	Park of Khuen-Hédervár mansion	*Platanus* × *hybrida* (front of the garden)	Individual elements	3	B	x	x	
25	36	Berkesz	Park of Vay mansion	*Aesculus hippocastanum*	Linear element (row of trees)	1	A	x	x	
26	37	Putnok	Park of Serényi mansion	*Fraxinus* *excelsior*	Individual	1	A	x		
27	38	Mihályi	Park of Dőry mansion	*Aesculus hippocastanum* (2)	Individual	1	A		x	x
	39	Mihályi	Park of Dőry mansion	*Pinus nigra*	Individual	2	B			x
28	40	Dénesfa	Park of Cziráky mansion	*Seauoiadendron giganteum*	Individual	3	B	x		x
	41	Dénesfa	Park of Cziráky mansion	*Fraxinus* *angustifolia*	Individual	1	A		x	
	42	Dénesfa	Park of Cziráky mansion	*Tilia cordata*	Individual	1	A		x	
	43	Dénesfa	Park of Cziráky mansion	*Larix decidua*	Individual	1	A		x	x

* Dead at the time of writing.
